# Oxidation resistance 1 functions in the maintenance of cellular survival and genome stability in response to oxidative stress-independent DNA damage

**DOI:** 10.1186/s41021-020-00168-w

**Published:** 2020-11-10

**Authors:** Ako Matsui, Kazunari Hashiguchi, Masao Suzuki, Qiu-Mei Zhang-Akiyama

**Affiliations:** 1 Laboratory of Stress Response Biology, Department of Zoology, Division of Biological Sciences, Graduate School of Science, Kyoto University, Kitashirakawa Oiwake-cho, Sakyo-ku, Kyoto, 606-8502 Japan; 2 Department of Experimental Oncology, Research Institute for Radiation Biology and Medicine, Hiroshima University, 1–2–3 Kasumi, Minami-ku, Hiroshima, 734–8553 Japan; 3 Department of Biochemistry, Fukuoka Dental College, 2-15-1 Tamura, Sawara-ku, Fukuoka, 814-0193 Japan; 4 Department of Basic Medical Sciences for Radiation Damages, National Institute of Radiological Sciences, National Institutes for Quantum and Radiological Science and Technology, 4-9-1 Anagawa, Inage-ku, Chiba, 263-8555 Japan

**Keywords:** OXR1, DNA damage response, Cell cycle checkpoint, Cellular survival, Protein expression

## Abstract

**Background:**

DNA damage is generated by various intrinsic and extrinsic sources such as reactive oxygen species (ROS) and environmental mutagens, and causes genomic alterations. DNA damage response (DDR) is activated to induce cell cycle arrest and DNA repair. Oxidation resistance 1 (OXR1) is a protein that defends cells against oxidative stress. We previously reported that OXR1 protein functions in the regulation of G2-phase cell cycle arrest in cells irradiated with gamma-rays, suggesting that OXR1 directly responds to DNA damage.

**Purpose:**

To clarify the functions of OXR1 against ROS-independent DNA damage, HeLa and OXR1-depleted HeLa cells were treated with heavy-ion beams and the ROS-independent DNA-damaging agent methyl methanesulfonate (MMS).

**Results:**

First, OXR1-depleted cells exhibited higher sensitivity to MMS and heavy-ion beams than control cells. Next, OXR1 depletion increased micronucleus formation and shortened the duration of G2-phase arrest after treatment with MMS or heavy-ion beams. These results suggest that OXR1 functions in the maintenance of cell survival and genome stability in response to DNA damage. Furthermore, the OXR1 protein level was increased by MMS and heavy-ion beams in HeLa cells.

**Conclusions:**

Together with our previous study, the present study suggests that OXR1 plays an important role in the response to DNA damage, not only when DNA damage is generated by ROS.

## Introduction

DNA damage is generated by various intrinsic and extrinsic sources such as reactive oxygen species (ROS) and environmental mutagens, and can cause genomic alterations, leading to cancer and neuronal diseases. Oxidative stress is the condition in which ROS are accumulated excessively [1, 2]. In most eukaryotes, Oxidation resistance 1 (OXR1) protein protects cells and organisms against oxidative stress [3]. The expression of endogenous OXR1 protein is induced under oxidative stress conditions in human cells [4, 5]. Previous studies suggested that OXR1 inhibits the generation of oxidative DNA damage by inhibiting oxidative stress to protect cellular survival and genomic integrity [6–11]. DNA damage response (DDR) induces cell cycle checkpoints and DNA damage repair system [1, 12]. Cell cycle checkpoints inhibit cell cycle progression and provide ample time for DNA repair. When DNA damage is sufficiently repaired, the cell cycle continues to progress. If the state of DNA damage exceeds repair capacity, cell death is provoked. Thus, DDR allows genomic stable cells to survive and prevents the proliferation of genomically unstable cells [1, 13, 14]. OXR1 is thought to affect the activation of G2-phase cell cycle checkpoint through oxidative stress inhibition [11].

Recently, we demonstrated that human OXR1 protein participates in genomic stability through the reduction of oxidative stress and in cell cycle checkpoint in cells irradiated with gamma-rays (γ-rays) [15]. In addition, the function of OXR1 in the regulation of G2-phase arrest was partially independent of oxidative stress. This phenomenon may be due to DNA strand breaks generated directly by depositing energy on the DNA strand and in addition to the indirect damage caused by ROS [16]. These results implied that OXR1 functions in the response to ROS-independent DNA damage.

DNA double-strand breaks (DSBs) are generated by both ROS-dependent and ROS-independent manner [1, 16]. Methyl methanesulfonate (MMS), one of DNA-alkylating reagents, methylates DNA bases, and the methylated sites are converted to AP sites and strand breaks in the process of DNA damage repair [1, 17]. Irradiation with heavy-ion beams, such as carbon- and iron-ion beams, at high linear energy transfer (LET) has biological effects mainly through ROS-independent mechanism, which is different from low LET irradiation, including γ-rays. Therefore, DNA damage generated by irradiation with heavy-ion beams are DSBs and more complex DNA lesions than low LET radiation [18–21]. Thus, irradiation with heavy-ion beams and treatment with MMS can generate DNA damage in a ROS-independent manner.

In the present study, to clarify the functions of OXR1 in response to ROS-independent DNA damage, we treated HeLa cells and OXR1-depleted HeLa cells with MMS and heavy-ion beams. Our study suggested that OXR1 plays an important role in the response to DNA damage.

## Materials and methods

### Cells and treatment

OXR1-depleted HeLa cells were established as described previously [15]. Cells were cultured in Dulbecco’s modified Eagle’s medium (low glucose, Wako Pure Chemical Industries) supplemented with 10% fetal bovine serum at 37 °C in a humidified incubator supplied with 5% CO_2_. Methyl methanesulfonate (MMS, Tokyo Chemical Industry) was dissolved in distilled water at the concentration of 1 M. Irradiation by heavy-ion beams was performed in the Heavy Ion Medical Accelerator in Chiba (HIMAC) at the National Institute of Radiological Sciences (NIRS) in Japan. Details of the treatment conditions are described in the results or figure legends.

### Colony formation assay

For MMS treatment, 100 cells were seeded in 60-mm diameter dishes and incubated for 5–6 h. The cells were constitutively treated with 80, 160 or 320 μM MMS. Plating efficiency was more than 90% in OXR1-depleted cells and control cells. For irradiation with heavy-ion beams, 500 or 1000 cells were seeded in T25 culture flasks (Falcon 3014 or CORNING 25 cm^2^ Triangular Angled Neck Cell Culture Flask) since the plating efficiency was around 30–50%, and incubated for 10–16 h. The cells were irradiated with carbon-ion beams (0, 2, 4 or 6 Gy, 290 MeV/nucleon, 87.0 keV/μm) or iron-ion beams (0, 1, 3 or 5 Gy, 500 MeV/nucleon, 200 keV/μm). After 10–14 days, the samples were stained with crystal violet dissolved in 20% methanol. The number of colonies containing more than 50 cells was counted.

### Cell synchronization

Cell synchronization to G1/S-phase was performed as described previously [15]. Cells were seeded in culture dishes and cultured for one or two days. The cells were treated with 2.5 mM thymidine (Wako Pure Chemical Industries) for 20–24 h, followed by incubation in fresh medium for 10 h. The cells were treated with 1 mM hydroxyurea (ACROS ORGANICS). After 14–16 h, the cells were synchronized to G1/S phase, and then exposed to DNA-damaging agents.

### Quantification of micronucleus (MN) formation

We used MN formation as biological endpoint for genotoxic effects and chromosomal instability [22]. In 35-mm diameter dishes, 0.7–0.8 × 10^5^ cells/dish were seeded and synchronized at G1/S-phase. The cells were treated with heavy-ion beams or MMS as described in the figure legends. After recovery incubation, the cells were fixed with PBS / 4% paraformaldehyde for 15 min at 4 °C and permeabilized with 0.1% Triton X-100 for 5 min at room temperature. Nuclei were stained with 5 μg/ml of DAPI for 5 min followed by washing with PBS. Micronuclei were observed using the fluorescence microscope OLYMPUS IX70 equipped with OLYMPUS DP50 using the 10 × or 20 × objective lens. The percentage of cells with micronuclei (≥ 460 cells per condition per experiment) was calculated.

### Cell cycle analysis

Cells were seeded in T25 flasks (Falcon) for irradiation or 60-mm diameter dishes for MMS treatment and cultured to approximately 20–40% confluency for one or two days. The cells were synchronized in G1/S-phase, followed by irradiation or MMS treatment. The cells were fixed with 70% ethanol at − 20 °C for more than 16 h. The cells were counter-stained with PBS/ 50 μg/ml of propidium iodide (PI, Sigma-Aldrich) containing 5 μg/ml of RNase. Fluorescence signal was detected by FACSCalibur (BD Biosciences) for 20,000 cells per sample and cell cycle distribution was analyzed using the BD CellQuest Pro Software (BD Biosciences).

### Western blot

Western blot was performed as described previously [15]. Briefly, whole cell lysates were separated by SDS-PAGE, followed by transfer to nitrocellulose membranes. The membranes were blocked with skim milk, and then proteins in the membranes were reacted with antibodies. Signals were developed using chemiluminescence (ECL, Amersham) and the membranes were then exposed to X-ray film (FUJIFILM). Images were analyzed by ImageJ 1.5a (Wayne Rasband National Institute of Health, USA http://imagej.nih.gov/ij). Antibodies used were: OXR1 (purified previously [15], 1:3000), Beta-actin (A5361, Sigma-Aldrich, 1:10,000), Beta-Tubulin (sc-9104, Santa Cruz Biotechnology, 1:1000), Rabbit-IgG-HRP (sc-2030, Santa Cruz Biotechnology, 1:5000) and Mouse-IgG-HRP (sc-2005, Santa Cruz Biotechnology, 1:5000).

### Statistical analysis

The data for the statistical assay were from more than three independent experiments. The data are presented as the mean ± s.e.m. or s.d.. Statistical differences between conditions were analyzed by ANOVA with the Welch’s, Student’s t-test or the Dunnett’s test using R 3.5.0 GUI 1.70 El Capitan build (7521), S. Urbanek, H.-J. Bibiko, & Stefano M. Iacus, See http://www.R-project.org for more information. *p*-values < 0.05 were considered significant.

## Results and discussion

### Sensitivity to MMS and heavy-ion beams

**Fig. 1:**
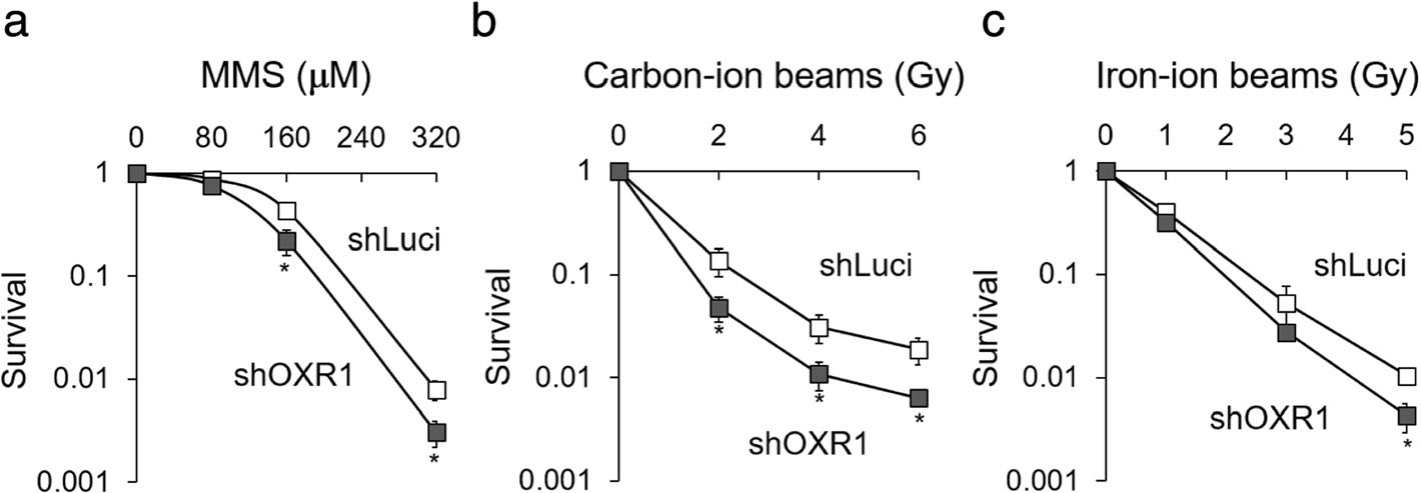
Sensitivity to MMS and heavy-ion beams. **a-c** Quantification of cellular survival. OXR1-depleted cells (shOXR1; closed square) and control cells (shLuci; open square) were (**a**) exposed constitutively to MMS (80, 160 or 320 μM) for 11 days, or irradiated with (**b**) carbon-ion beams (0, 2, 4 or 6 Gy, 290 MeV/nucleon, 87.0 keV/μm) or (**c**) iron-ion beams (0, 1, 3 or 5 Gy, 500 MeV/nucleon, 200 keV/μm). Cellular survival was analyzed by the colony formation assay. Means ± s.d. of *n* = three independent experiments, * *p* < 0.05, two-tailed Student’s t-test

To investigate whether OXR1 defends against ROS-independent DNA damage, cells were treated with MMS or irradiated with heavy-ion beams and the cell viability was evaluated by colony formation assay. OXR1-depleted cells exhibited significantly higher sensitivity to MMS treatment, carbon-ion beams and iron-ion beams irradiation than control cells (Fig. 1a-c). These results suggested that OXR1 plays an important role in defends against ROS-independent DNA damage, which is mainly consists of DNA strand breaks.

The previous reports and our recent study demonstrated that depletion of OXR1 exhibited increased sensitivity to oxidative stress, indicating that OXR1 functions in protection of cells from genotoxic oxidative stress [5, 9, 15]. Together with present study, OXR1 maintains cellular survival in the response to DNA damage, and the source of DNA damage activating OXR1 is not limited to ROS.

### MN formation and cell cycle distribution

Recently our study demonstrated that OXR1 depletion increases MN formation and accumulation of cells in G2/M-phase after treatment with hydrogen peroxide or irradiation with γ-rays to the synchronized cells in G1/S-phase [15]. We found that the increased level of MN formation in OXR1-depleted cells were caused by both ROS-dependent and -independent factors [15]. However, the generation of MN through ROS-independent mechanism needs further verification. To investigate whether OXR1 depletion also induces MN formation actually by ROS-independent DNA damage, the synchronized cells in G1/S-phase were irradiated with heavy-ion beams, and the number of cells containing MN was measured.

Cells were irradiated with heavy-ion beams at the dose in which 1% of survival rate was detected following our previous experiment [15]. We observed MN formation and cell cycle distribution until 24 h after irradiation with γ-rays in previous report [15]. Since it has been reported that the duration of G2-phase arrest in the cells irradiated with heavy-ion beams is longer than low LET radiation [23, 24], we decided to observe MN formation in irradiated cells over a period of time longer than 24 h after irradiation.

**Fig. 2:**
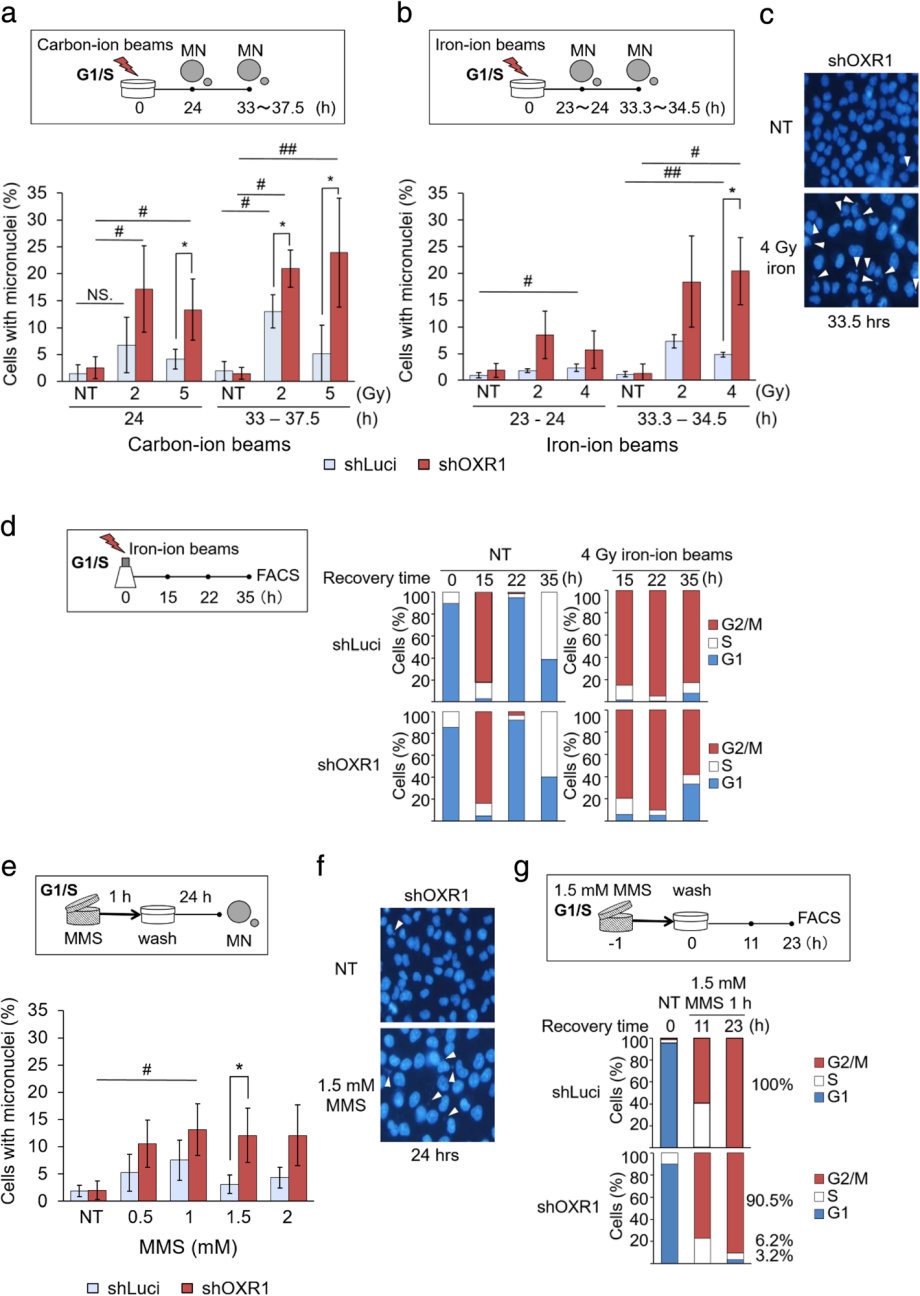
MN formation level after treatment with MMS and heavy-ion beams. **a-c** Quantification of the MN formation level. OXR1-depleted HeLa cells or control cells were synchronized at G1/S-phase. The cells were irradiated with (**a**) carbon-ion beams (2 or 5 Gy, 290 MeV/nucleon, 83.8–86 keV/μM) or (**b**) iron-ion beams (2 or 4 Gy, 500 MeV/nucleon, 200 keV/μm). **c** Representative fluorescent images of DAPI-stained nuclei. Arrowheads indicate micronuclei. **d** Distribution of the cell cycle. Cells were synchronized at G1/S phase. The cells were irradiated with 4 Gy of iron-ion beams (500 MeV/nucleon, 200 keV/μm). After the indicated times, cell cycle distributions were measured by FACS. NT: no treatment. **e** Quantification of the MN formation level in cells treated with MMS (0.5, 1, 1.5 or 2 mM) for 1 h. **f** Representative fluorescent images of DAPI-stained nuclei. **g** Distribution of the cell cycle in cells treated with 1.5 mM MMS for 1 h. The percentage of cells with micronuclei was calculated (a, b ≥ 1000, d. ≥ 460 per condition per experiment). Means ± s.e.m. of *n* = three independent experiments, # *p* < 0.05, ## *p* < 0.01, three-way ANOVA with Dunnett’s multiple comparisons test, * *p* < 0.05, two-tailed Welch’s and Student’s t-test. NT, no treatment

At 24 h or 33–37.5 h after irradiation with 5 Gy of carbon-ion beams, MN formation level in OXR1-depleted cells was significantly increased compared with that in control cells (Fig. 2a). Similarly, at 33.3–34.5 h after irradiation with 4 Gy of iron-ion beams, the level of MN formation in OXR1-depleted cells was higher than that in control cells (Fig. 2b, c). In cells in which MN formation is accelerated, more DNA lesions are unrepaired [22, 25]. We next analyzed the cell cycle distribution of the cells synchronized G1/S-phase, followed by irradiation with iron-ion beams. At 35 h after irradiation with 4 Gy of iron-ion beams, a smaller fraction of OXR1-depleted cells in G2/M-phase was observed compared with control cells (Fig. 2d). In the case of MMS treatment, higher MN formation level and a smaller fraction of G2/M-phase were observed in OXR1-depleted cells (Fig. 2e-g), as similar to iron-ion beam treatment.

Less distribution of cells in G2/M-phase compared with control and untreated cells most likely indicates partial defects of G2-phase checkpoint control, which is one of DNA damage checkpoint control [26]. If the G2-phase checkpoint control is insufficient, ROS-independent DNA strand breaks generated by heavy-ion beams and MMS might not be fully repaired, leading to genetic aberration and cell death. Taken together, our results suggest that OXR1 participates in genomic stability through regulation of the G2-phase checkpoint.

The main mechanism by which MMS treatment damages DNA is alkylation, not oxidation by ROS [17, 27]. In cells irradiated with heavy-ion beams, the effects of ROS-independent factors on cellular components, including DNA, have been thought to be larger than those of ROS [21, 28–31]. Thus, MMS treatment and heavy-ion beam irradiation provoke mainly ROS-independent damage. Furthermore, we previously showed that the depletion of OXR1 suppresses the G2-phase checkpoint, ROS-independently [15]. Together with the previous studies, the present study shows that OXR1 functions in the response to DNA damage generated in a ROS-independent manner.

### Protein level of OXR1

**Fig. 3:**
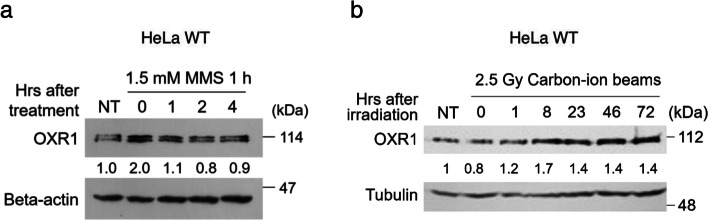
Induction of OXR1 protein level. **a, b** OXR1 protein level. HeLa cells (indicated as HeLa WT) were treated with (**a)** 1.5 mM MMS for 1 h or (**b**) 2.5 Gy of carbon-ion beams, and the cells were incubated for the indicated times. The OXR1 protein level in crude extracts was assessed by Western blot using anti-OXR1 antibody. Proteins were separated with 10% SDS-PAGE gel. OXR1 protein was detected as (**a**) two or (**b**) single bands because of the difference in each gel running time. It has been confirmed that both two bands are OXR1 in our previous study [15]. Relative OXR1 protein levels (vs. no treatment) normalized by beta-actin or tubulin are shown below OXR1 blots. The size of marker proteins is shown on the right of the image. Images were analyzed by Image J software. NT, no treatment

To investigate whether the protein level of OXR1 is increased by ROS-independent genotoxic stresses, HeLa cells were treated with 1.5 mM MMS for 1 h or 2.5 Gy of irradiated with carbon-ion beams, and the protein level of endogenous OXR1 was analyzed by Western blot. The experiment was repeated twice and a representative blot is shown in Fig. 3. The OXR1 protein level increased to approximately two-fold just after MMS treatment and decreased to a steady-state level 4 h after treatment (Fig. 3a). When HeLa cells were irradiated with carbon-ion beams, OXR1 protein level reached a maximum by 8 h after irradiation, and this higher protein level continued until at least 72 h after irradiation (Fig. 3b).

Compared with MMS-treatment, delayed elevation of OXR1 protein level was observed in carbon-ion beams irradiation. This might be due to that irradiation of heavy-ion beams produce more complex DNA damage than MMS-treatment. Thus, the expression of OXR1 protein was induced by both MMS and carbon-ion beams. One of possibility is that increased amount of OXR1 protein might require for maintenance of G2-phase checkpoint control. So far, it was known that the protein level of OXR1 is induced by the oxidative stress [4, 5]. This study revealed that the OXR1 protein level is also increased by treatment with MMS or heavy-ion beams, suggesting that OXR1 protein is involved in the regulation of DDR (Fig. 2), even when the DNA damage is not triggered by oxidative stress.

## Conclusions

In this study, we found that the OXR1 depletion increased cell sensitivity and the OXR1 protein level is increased in response to ROS-independent DNA damage. OXR1 protein helps maintain genome stability directly against ROS-dependent and independent DNA damage.

## Data Availability

All data generated or analyzed during this study are included in this published article.

## Abbreviations

ROS: Reactive oxygen species
DDR: DNA damage response
OXR1: Oxidation resistance 1
MMS: Methyl methanesulfonate
MN: Micronucleus
LET: Linear energy transfer
DSB: Double strand-break
